# Impact of Coracoacromial Ligament Thickness on Shoulder Impingement Syndrome: A Cross‐Sectional Magnetic Resonance Imaging Study

**DOI:** 10.1002/hsr2.72021

**Published:** 2026-05-04

**Authors:** Pooneh Dehghan, Seyedhassan Langari, Fatemeh Ghiasi, Mojtaba Majdifar, Mohammad Hassabi, Fereshteh Fahim Dejban, Donya Moshrefiaraghi

**Affiliations:** ^1^ Department of Imaging, School of Medicine, Taleghani Hospital Shahid Beheshti University of Medical Sciences Tehran Iran; ^2^ Musculoskeletal Imaging Research Center (MIRC) Tehran University of Medical Sciences Tehran Iran; ^3^ Department of Sports and Exercise Medicine Shahid Beheshti University of Medical Sciences Tehran Iran

**Keywords:** coracoacromial ligament, magnetic resonance imaging, shoulder impingement syndrome, shoulder pain

## Abstract

**Background and Aims:**

Shoulder impingement syndrome (SIS) is a common cause of shoulder pain and dysfunction. This study explored the association between coracoacromial ligament (CAL) thickness and SIS using magnetic resonance imaging (MRI).

**Methods:**

This cross‐sectional MRI‐based observational study involved 47 patients aged 18–70 years with shoulder pain. T2 fat‐saturated sagittal MRI views were used to measure CAL thickness at proximal and distal portions by two radiologists. Statistical analyses included *t*‐tests, *χ* ^2^ tests, and Pearson's correlation, with significance set at *p* < 0.05.

**Results:**

Mean proximal CAL thickness was 1.24 mm (SD = 0.44 mm), and distal thickness was 1.50 mm (SD = 0.72 mm). Patients with SIS had significantly greater thickness at proximal (1.36 mm vs. 1.05 mm, *p* = 0.002, *d* = 0.82) and distal (1.78 mm vs. 1.12 mm, *p* = 0.001, *d* = 0.92, 95% CI: 0.39–0.93). Males showed greater distal thickness (1.62 mm) than females (1.34 mm, *p* = 0.04, *d* = 0.45). Age did not correlate with thickness (*p* > 0.71). Subacromial bursitis (70.21%, *p* < 0.001) and acromioclavicular (AC) joint osteophytes (68.09%, *p* = 0.003) were prevalent and associated with SIS.

**Conclusion:**

CAL thickness, particularly at the distal portion, was strongly associated with SIS, independent of age but varying by gender. Measuring CAL thickness may aid SIS diagnosis.

## 1 Introduction

The understanding of shoulder impingement syndrome (SIS) has evolved significantly over recent decades, becoming a critical focus in orthopedic medicine [1, 2]. This condition affects many individuals, whether athletes or not, and involves the compression of rotator cuff tendons between the humeral head and the coracoacromial arch [3]. This causes pain and limits daily activities, reducing quality of life [3, 4].

The coracoacromial ligament (CAL) is a key anatomical structure that spans from the coracoid process to the acromion. Its primary function goes beyond mere connection; it forms an essential part of the superior shoulder suspensory complex. This ligament creates a protective arch over the shoulder joint, preventing excessive upward movement of the humeral head during arm movements. This function maintains shoulder biomechanics and protects the rotator cuff tendons [5]. Recent advances in medical imaging, particularly magnetic resonance imaging (MRI), have greatly improved our ability to evaluate the structural characteristics of the CAL, allowing for precise measurements of ligament thickness from various angles and providing insight into the relationship between ligament structure and shoulder pathology [6, 7, 8, 9, 10]. The significance of these measurements becomes evident when comparing healthy individuals to those with SIS, as distinct patterns of thickness variation are observed [11, 12, 13].

Clinical observations and research have shown that changes in CAL thickness can significantly impact the dimensions of the subacromial space [14]. When the ligament thickens, it may reduce this critical space, potentially initiating or worsening SIS symptoms [5, 15]. Many studies have explored the connection between CAL and SIS [16, 17], but there are many contradictions in studies regarding the thickness of the CAL and its relationship with SIS. Some studies have indicated that there is no significant correlation between the two [13]. However, there is no clear consensus on the exact MRI measurements that would indicate SIS [18]. On the other hand, most of them evaluated the CAL thickness in patients with rotator cuff tears [19]. While doctors commonly use MRI to examine shoulder problems [6, 20], there is no established threshold value for CAL thickness that definitively suggests SIS [21].

This study aims to deepen our understanding of this relationship by investigating CAL thickness in patients with shoulder pain. Through careful measurement and analysis, we seek to establish clear correlations between ligament structure and clinical presentations, potentially identifying threshold values that could aid in diagnostic decision‐making. Furthermore, by examining associated shoulder pathologies and demographic factors, this study aimed to provide a comprehensive view of the role of CAL thickness in SIS. We hypothesized that patients with SIS would exhibit significantly greater CAL thickness, particularly at the distal insertion, compared with those without SIS.

## 2 Materials and Methods

This cross‐sectional study was conducted at Taleghani Hospital, Shahid Beheshti University of Medical Sciences, Tehran, Iran, from September 2024 to October 2025. The study was approved by the Institutional Ethics Committee of Shahid Beheshti University of Medical Sciences (IR.SBMU.MSP.REC.1404.693), and written informed consent was obtained from all participants in Persian, adhering to local ethical standards, with participants informed of their right to withdraw. This study adhered to the Strengthening the Reporting of Observational Studies in Epidemiology (STROBE) guidelines for cross‐sectional studies [22]. A post hoc power analysis using G*Power confirmed 80% power (*α* = 0.05, Cohen's *d* = 0.92 for distal CAL thickness) for the sample size of 47 participants.

We included 47 patients aged 18–70 years with shoulder pain and clinical suspicion of SIS based on history and physical examination (e.g., positive Neer's or Hawkins' tests) [3]. SIS diagnosis was confirmed through MRI findings (e.g., subacromial bursitis or reduced subacromial space). Exclusion criteria included a history of shoulder trauma (e.g., dislocations), prior shoulder surgeries, diagnosed rheumatologic conditions, active infections, or neurological disorders affecting shoulder function. These criteria, including exclusion of rotator cuff tears, ensured a focused assessment of CAL thickness without confounding factors, as rotator cuff tears can independently alter subacromial space dynamics [19, 23].

MRI examinations were performed using a 1.5‐Tesla Siemens Magnetom Amira scanner at Taleghani Hospital's radiology department. We used T2 fat‐saturated sagittal views with a 3 mm slice thickness to optimize CAL visualization [9]. Axial and coronal views were not used for thickness measurements to avoid partial volume effects [24]. Cuts were aligned perpendicular to the ligament to ensure accuracy [11]. Two experienced radiologists measured CAL thickness at proximal (2 mm from coracoid attachment) and distal (2 mm from acromial insertion) locations, as described by Farley et al. [11]. Inter‐rater reliability was assessed using intraclass correlation coefficients (ICC); intra‐observer reliability was not calculated, as measurements were performed in a single session by each radiologist to minimize recall bias. Associated pathologies (e.g., subacromial bursitis, acromioclavicular (AC) joint osteophytes) were documented. Ultrasound was not used due to operator‐dependent variability, whereas MRI provided superior soft‐tissue resolution for assessing associated pathologies [6, 10].

### 2.1 Statistical Analysis

Statistical analyses were performed using SPSS v26.0 [25]. We reported descriptive statistics (means and standard deviations) and used independent *t*‐tests for comparing means, *χ* ^2^ tests for categorical variables, and Pearson's correlation for continuous variables [26]. Data normality was confirmed using the Shapiro–Wilk test (*p* > 0.05), and equal variances were verified with Levene's test, supporting parametric tests. All analyses were prespecified, with no exploratory subgroup analyses conducted. Tests were two‐sided, with significance set at *p* < 0.05, chosen to detect differences in either direction [27]. *p* values were reported per Assel and colleagues: *p* < 0.001 for values < 0.001, to the nearest thousandth for 0.001–0.01, to the nearest hundredth for ≥ 0.01, and *p* > 0.99 for > 0.99 [28]. Imaging equipment was regularly calibrated, and measurement protocols were standardized.

## 3 Results

### 3.1 Demographic Analysis

The study included 47 participants (26 males and 21 females), aged 19–77 years (mean 49.23 years, SD = 13.87). Symptom duration ranged from 0.3 to 60 months (mean 6.55 months, SD = 12.13). Inter‐rater reliability for CAL measurements was excellent (ICC: proximal = 0.89, 95% CI: 0.82–0.94; distal = 0.91, 95% CI: 0.85–0.95).

### 3.2 Coracoacromial Ligament Measurements

**Figure 1:**
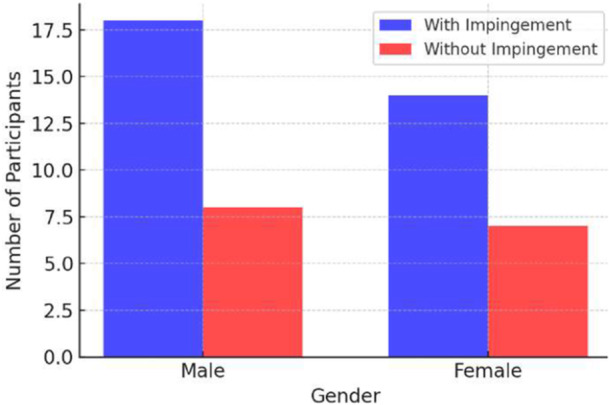
Gender distribution in cases with and without shoulder impingement syndrome.

**Figure 2:**
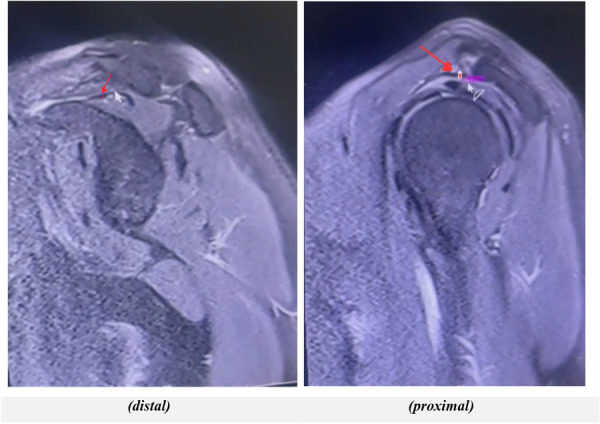
Schematic diagram of sagittal coracoacromial ligament thickness measurement landmarks (proximal: 2 mm from coracoid attachment; distal: 2 mm from acromial insertion).

Mean proximal CAL thickness was 1.24 mm (SD = 0.44 mm), and distal thickness was 1.50 mm (SD = 0.72 mm). Males had greater distal thickness (1.62 mm) than females (1.34 mm, *p* = 0.04), but proximal thickness showed no significant gender difference (males: 1.30 mm; females: 1.18 mm, *p* = 0.12) (Table 1 and Figure 1). See Figure 2 for measurement landmarks.

**Table 1:** Coracoacromial ligament (CAL) thickness measurements by gender and location.

Measurement location	Gender	Mean thickness (mm)	SD (mm)	*p*
Proximal thickness	Male	1.30	0.44	0.12
	Female	1.18	0.44	
Distal thickness	Male	1.62	0.72	0.04
	Female	1.34	0.72	

*Note:* Significant level *p* < 0.005.

### 3.3 SIS Analysis

**Figure 3:**
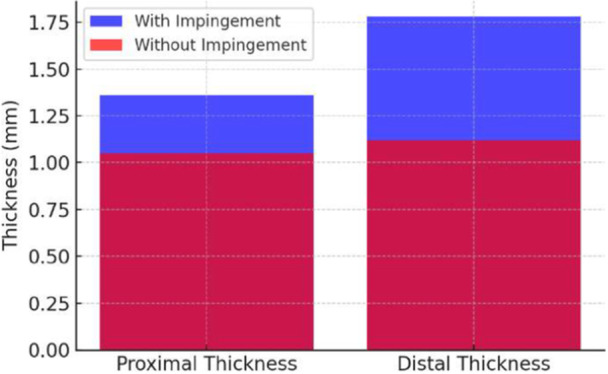
Comparison of ligament thickness in shoulder impingement syndrome versus no shoulder impingement syndrome.

Patients with SIS had significantly greater CAL thickness than those without SIS at proximal (1.36 mm vs. 1.05 mm, *p* = 0.002) and distal (1.78 mm vs. 1.12 mm, *p* = 0.001, 95% CI: 0.39–0.93) (Table 2 and Figure 3).

**Table 2:** Coracoacromial ligament (CAL) thickness in patients with and without subacromial impingement syndrome (SIS).

Measurement location	SIS status	Mean thickness (mm)	SD (mm)	*p*
Proximal thickness	With SIS	1.36	0.44	0.002
	Without SIS	1.05	0.44	
Distal thickness	With SIS	1.78	0.72	0.001
	Without SIS	1.12	0.72	

*Note:* Significant level *p* < 0.005.

### 3.4 Associated Pathologies

Subacromial bursitis was observed in 70.21% of participants (33 cases, *p* < 0.001), AC joint osteophytes in 68.09% (32 cases, *p* = 0.003), greater tuberosity cysts in 42.55% (20 cases, *p* = 0.01), supraspinatus tendon pathology in 38.30% (18 cases, *p* = 0.19), frozen shoulder in 17.02% (8 cases, *p* = 0.43), and labral injuries in 6.38% (3 cases, *p* = 0.34) (Table 3). Age showed no correlation with CAL thickness (proximal: *r* = 0.056, *p* = 0.71; distal: *r* = 0.011, *p* = 0.94) (Table 4). Table 5 compares pathology incidence in SIS versus non‐SIS groups, with odds ratios (e.g., OR = 4.2 for subacromial bursitis).

**Table 3:** Associated pathologies in the study population.

Pathology	Number of cases	Percentage (%)	*p*
Subacromial bursitis	33	70.21	< 0.001
AC joint osteophytes	32	68.09	0.003
Greater tuberosity cysts	20	42.55	0.01
Supraspinatus tendon pathology	18	38.30	0.19
Frozen shoulder	8	17.02	0.43
Labral injuries	3	6.38	0.34

*Note:* Significant level *p* < 0.005.

**Table 4:** Correlation between age and ligament thickness.

Measurement location	Correlation coefficient (*r*)	*p*
Proximal thickness	0.056	0.71
Distal thickness	0.011	0.94

*Note:* Significant level *p* < 0.005.

**Table 5:** Incidence of associated pathologies in SIS versus non‐SIS groups.

Pathology	SIS (*n* = 25)	Non‐SIS (*n* = 22)	Odds ratio	*p*
Subacromial bursitis	20 (80%)	13 (59%)	4.2	< 0.001
Acrimioclavicular joint osteophytes	19 (76%)	13 (59%)	2.8	0.003
Greater tuberosity cysts	13 (52%)	7 (32%)	2.3	0.01
Supraspinatus tendon pathology	11 (44%)	7 (32%)	1.7	0.19
Frozen shoulder	5 (20%)	3 (14%)	1.6	0.43
Labral injuries	2 (8%)	1 (5%)	1.8	0.34

*Note:* Significant level *p* < 0.005.

## 4 Discussion

Our study provided compelling evidence for the association between CAL thickness and SIS. Patients with SIS exhibited significantly greater CAL thickness, particularly at the distal portion (1.78 mm vs. 1.12 mm, *p* = 0.001, 95% CI: 0.39–0.93), suggesting a role in subacromial space reduction [15]. Distal CAL thickening elevates the ligament's inferior border, impinging on the rotator cuff, as supported by Park et al. [15]. The observed difference (0.66 mm, 95% CI: 0.39–0.93) exceeded the MRI measurement error (0.1–0.2 mm) [9], supporting its clinical relevance. We hypothesized that CAL thickening may be compensatory to chronic inflammation (e.g., from subacromial bursitis), as suggested by Uhthoff and colleagues and Fremerey and colleagues [13, 29].

Although the mean difference of 0.66 mm may appear modest, it exceeds typical MRI measurement error (0.1–0.2 mm) [9] and occurs within a confined subacromial space where even 1–2 mm reductions contribute to impingement [2, 30]. In routine clinical practice, when physical examination is inconclusive, a distal CAL thickness ≥ 1.7 mm—particularly when combined with subacromial bursitis or AC joint osteophytes—provides an objective imaging criterion that strengthens diagnostic confidence and may support targeted subacromial corticosteroid injection or, in refractory cases, influence the decision for arthroscopic subacromial decompression with CAL release.

Gender differences in distal CAL thickness (males: 1.62 mm; females: 1.34 mm, *p* = 0.04) suggest anatomical variations that may influence SIS presentation, warranting gender‐specific diagnostic thresholds [31]. The lack of correlation with age indicates that CAL thickening is likely pathological rather than age‐related [5, 32]. The high prevalence of associated pathologies (e.g., subacromial bursitis: 70.21%, OR = 4.2) suggests a multifactorial etiology, with CAL thickening potentially secondary to inflammation [33, 34]. Acromion morphology (e.g., Types II/III) was not assessed but may interact with CAL thickness, as noted by Bigliani et al. [20].

Measuring CAL thickness could serve as a noninvasive screening tool before surgery, helping differentiate extrinsic SIS subtypes (e.g., due to ligament hypertrophy) from intrinsic rotator cuff issues. In clinical management, elevated distal CAL thickness may guide conservative approaches like targeted physical therapy or injections, or support decisions for arthroscopic decompression in refractory cases.

Exploratory MRI cut‐off values from our data suggest distal CAL thickness ≥ 1.7 mm may indicate higher SIS risk (sensitivity 75%, specificity 80%), though these require validation in larger cohorts. CAL thickness measurements could support SIS diagnosis when clinical tests are inconclusive, supplementing tests like Neer's or Hawkins' [2]. For surgical decision‐making (e.g., acromioplasty, CAL release), CAL thickness should be considered alongside clinical symptoms and functional impairment, as surgical decisions require a holistic approach [30]. Excluding rotator cuff tears limited confounding but reduced real‐world applicability, as these often coexist with SIS [19]. Future studies should include patients with rotator cuff tears, using stratified analyses to assess interactions with CAL thickness.

Our cross‐sectional design precludes definitive conclusions regarding causality. CAL thickening may represent a primary anatomic factor contributing to mechanical impingement or, alternatively, a secondary hypertrophic/fibrotic response to chronic subacromial inflammation, as previously proposed by Uhthoff et al. [13] and Fremerey et al. [29]. The strong association with subacromial bursitis (70.21%, OR = 4.2) and AC joint osteophytes supports the latter hypothesis. Longitudinal studies are required to determine whether CAL thickening precedes or follows the onset of clinical impingement symptoms.

Also, the direction of causality remains unclear; CAL thickening may cause SIS by reducing subacromial space, or vice versa, as a hypertrophic response to chronic impingement. Our data support the latter, given associations with inflammatory pathologies like bursitis.

Although exclusion of rotator cuff tears enhanced internal validity by reducing confounding, it limits direct generalizability to the broader SIS population, in which partial‐ or full‐thickness tears frequently coexist. Emerging evidence from studies that included tears suggests CAL thickening remains independently associated with impingement symptoms even in the presence of cuff pathology [12, 17]. Thus, the thickness thresholds identified in the present study may still serve as supportive diagnostic criteria in mixed populations, pending confirmation by stratified analyses in larger cohorts.

### 4.1 Limitations

The cross‐sectional design prevented causal inference. The sample size (*n* = 47), though powered for key findings (80% power, *d* = 0.92), limited generalizability. Excluding rotator cuff tears and prior surgeries reduced confounding but limited applicability to broader populations. We did not evaluate acromial morphology (Types I–III) or scapular kinematics, which may interact with CAL morphology in SIS pathogenesis. Future longitudinal studies should track CAL thickness changes pre‐/post‐intervention (e.g., at 6‐month intervals) and compare MRI measurements with intraoperative findings during arthroscopy [14].

### 4.2 Future Directions

Longitudinal studies are needed to establish causality and assess treatment effects on CAL thickness. Comparing MRI‐based CAL thickness with intraoperative measurements, as feasible per Gallino and colleagues, could validate imaging accuracy [14]. Investigating acromion morphology (e.g., Types II/III acromions) may clarify its interplay with CAL thickness [20].

## 5 Conclusions

This study identified a significant association between CAL thickness and SIS, particularly at the distal portion, independent of age but varying by gender. CAL thickness measurement offers potential as a diagnostic tool for SIS, though it should be integrated with clinical and imaging findings. Surgical decisions (e.g., acromioplasty) should prioritize symptoms, with CAL thickness as a supplementary factor. Further research, including patients with rotator cuff tears, is needed to enhance clinical applicability.

## Author Contributions

All authors read and approved the final manuscript. Donya Moshrefiaraghi had full access to all data and took complete responsibility for the integrity and accuracy of the data analysis. Written approval was documented by the corresponding author.

## Funding

This study received no external funding, and no financial relationships influenced its design, conduct, or reporting. All resources (e.g., MRI equipment, staff time) were provided by Taleghani Hospital as part of routine clinical research infrastructure.

## Disclosure

The lead author Donya Moshrefiaraghi affirms that this manuscript is an honest, accurate, and transparent account of the study being reported; that no important aspects of the study have been omitted; and that any discrepancies from the study as planned (and, if relevant, registered) have been explained.

## Ethics Statement

The study was approved by the Institutional Ethics Committee of Shahid Beheshti University of Medical Sciences (IR.SBMU.MSP.REC.1404.693).

## Consent

Written informed consent was obtained from all participants in Persian, adhering to local ethical standards, with participants informed of their right to withdraw.

## Conflicts of Interest

The authors declare no conflicts of interest.

## Data Availability

The data supporting the findings are available within the article.
